# Supplementary figures and images for: Risk-guided maternity care to enhance maternal empowerment postpartum: A cluster randomized controlled trial
Source: PLoS One. 2020 Nov 20;15(11):e0242187. doi: 10.1371/journal.pone.0242187 (PMC7679010; doi:10.1371/journal.pone.0242187)

**S1 Fig: The timeline cluster diagram**


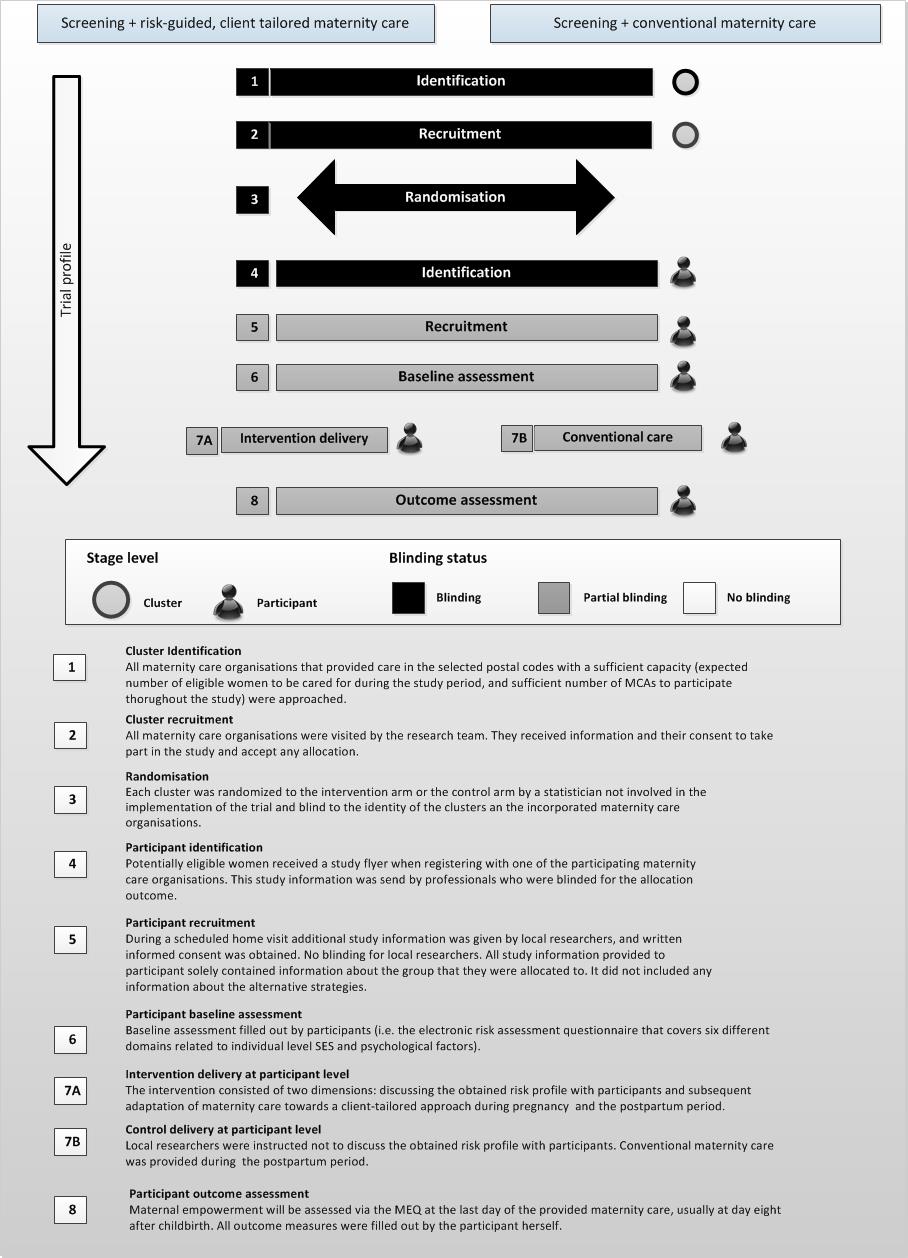

Supplement: S1 Fig — (DOCX) [file pone.0242187.s001.docx]
