# Supplementary material for: Risk-guided maternity care to enhance maternal empowerment postpartum: A cluster randomized controlled trial
Source: PLoS One. 2020 Nov 20;15(11):e0242187. doi: 10.1371/journal.pone.0242187 (PMC7679010; doi:10.1371/journal.pone.0242187)
Supplement: S1 Table — (DOCX) [file pone.0242187.s002.docx]

| Outcomes  **S1 Table: Outcome measures at participant level with definitions** | Postpartum period |  |
| --- | --- | --- |
|  | **Early (1-2 weeks after childbirth)** | **Late (6-12 weeks after childbirth)** |
| Primary | ***Maternal empowerment*** |  |
|  | Low empowerment score (no/yes) defined as a MEQ-score beneath the 20th centile within the control group |  |
| Secondary | ***Maternal health related quality of life*** | ***Maternal depression (postnatal depression)*** |
|  | Continuous score ranging from zero (dead) to one (full health) based on the validated EQ-5D-5L calculator | Dichotomous outcome based on the EPDS-sum score: "No" sum score <13 and "Yes" sum score >12. |
|  | ***Maternal perceived health*** | ***Maternal health care utilisation*** |
|  | Continuous score ranging from zero (the worst health possible) to 100 (the best health possible) based on the EuroQol-visual analogue scales (EQ-VAS) | Categorical outcome ranging from “No additional care” to "Admission in a hospital", based on a single question |
|  |  | ***Neonatal health care utilization*** |
|  |  | Categorical outcome ranging from: “No additional care” to "Admission in a hospital", based on a single question |
|  | ***Maternal cigarette use*** | ***Maternal cigarette use*** |
|  | Dichotomous outcome based on a single question | Dichotomous outcome based on a single question |
|  | ***Maternal alcohol use*** | ***Maternal alcohol use*** |
|  | Dichotomous outcome based on a single question | Dichotomous outcome based on a single question |
|  | ***Maternal drugs use*** | ***Maternal drugs use*** |
|  | Dichotomous outcome based on a single question | Dichotomous outcome based on a single question |

All predefined, individual outcome measures at the participant level with timing of assessment. The early postpartum period was defined as the period between seven through 14 days after childbirth, and the late postpartum period between six through twelve weeks after childbirth.
