# Supplementary material for: Risk-guided maternity care to enhance maternal empowerment postpartum: A cluster randomized controlled trial
Source: PLoS One. 2020 Nov 20;15(11):e0242187. doi: 10.1371/journal.pone.0242187 (PMC7679010; doi:10.1371/journal.pone.0242187)
Supplement: S2 Table — (DOCX) [file pone.0242187.s003.docx]

**S2 Table: baseline characteristics for participants who were lost to-follow up by timing of follow-up (early and late postpartum period)**

|  | Missing information participants | | | | | | | |
| --- | --- | --- | --- | --- | --- | --- | --- | --- |
|  | **Early postpartum period** | | | | **Late postpartum period** | | | |
|  | **No (n=799)** | | **Yes (n=780)** | | **No(n=1,105)** | | **Yes (n=474)** | |
|  |  |  |  |  |  |  |  |  |
| Maternal age | 31.27 | (4.7) | 31.87 | (4.5) | 31.21 | (4.8) | 32.38 | (4.1) |
| Parity |  |  |  |  |  |  |  |  |
| Primiparous | 411 | 51.4% | 395 | 50.6% | 548 | 49.6% | 258 | 54.4% |
| Multiparous | 388 | 48.6% | 385 | 49.4% | 557 | 50.4% | 216 | 45.6% |
| Cohabitation partners |  |  |  |  |  |  |  |  |
| Single | 39 | 4.9% | 12 | 1.5% | 41 | 3.7% | 10 | 2.1% |
| Living together | 760 | 95.1% | 768 | 98.5% | 1,064 | 96.3% | 464 | 97.9% |
| Immigrant status |  |  |  |  |  |  |  |  |
| Non-immigrant | 472 | 59.8% | 591 | 76.4% | 695 | 62.9% | 368 | 77.6% |
| First generation | 138 | 17.5% | 94 | 12.1% | 191 | 17.3% | 41 | 8.6% |
| Second generation | 179 | 22.7% | 89 | 11.5% | 210 | 19.0% | 58 | 12.2% |
| Missing | 10 | 1.3% | 6 | 0.8% | 9 | 0.8% | 7 | 1.5% |
| Health insurance |  |  |  |  |  |  |  |  |
| No | 0 | 0.0% | 2 | 0.3% | 0 | 0.0% | 2 | 0.4% |
| Yes | 799 | 100.0% | 778 | 99.7% | 1,105 | 100.0% | 472 | 99.6% |
| Education |  |  |  |  |  |  |  |  |
| Lower | 54 | 6.8% | 39 | 5.0% | 77 | 7.0% | 16 | 3.4% |
| Intermediate | 533 | 66.7% | 433 | 55.5% | 705 | 63.8% | 261 | 55.1% |
| High | 212 | 26.5% | 308 | 39.5% | 323 | 29.2% | 197 | 41.6% |
| Household income (euro/month) |  |  |  |  |  |  |  |  |
| <1500 | 132 | 16.5% | 58 | 7.4% | 164 | 14.8% | 26 | 5.5% |
| 1500-3000 | 317 | 39.7% | 281 | 36.0% | 433 | 39.2% | 165 | 34.8% |
| >3000 | 350 | 43.8% | 441 | 56.5% | 508 | 46.0% | 283 | 59.7% |
| Paid job during pregnancy |  |  |  |  |  |  |  |  |
| No | 211 | 26.4% | 135 | 17.3% | 288 | 26.1% | 58 | 12.2% |
| Yes | 588 | 73.6% | 645 | 82.7% | 817 | 73.9% | 416 | 87.8% |
| Neighbourhood deprivation |  |  |  |  |  |  |  |  |
| No | 432 | 54.1% | 455 | 58.3% | 619 | 56.0% | 268 | 56.5% |
| Yes | 367 | 45.9% | 325 | 41.7% | 486 | 44.0% | 206 | 43.5% |
| Smoking |  |  |  |  |  |  |  |  |
| No | 681 | 85.2% | 683 | 87.6% | 942 | 85.2% | 422 | 89.0% |
| Yes | 118 | 14.8% | 97 | 12.4% | 163 | 14.8% | 52 | 11.0% |
| Alcohol |  |  |  |  |  |  |  |  |
| No | 656 | 82.1% | 602 | 77.2% | 884 | 80.0% | 374 | 78.9% |
| Yes | 143 | 17.9% | 178 | 22.8% | 221 | 20.0% | 100 | 21.1% |
| Drugs |  |  |  |  |  |  |  |  |
| No | 783 | 98.0% | 773 | 99.1% | 1,084 | 98.1% | 472 | 99.6% |
| Yes | 16 | 2.0% | 7 | 0.9% | 21 | 1.9% | 2 | 0.4% |

Mean* with SD or number with % (presented as percentage of non-missing values). Missing value percentage of total.
