# Supplementary material for: Risk-guided maternity care to enhance maternal empowerment postpartum: A cluster randomized controlled trial
Source: PLoS One. 2020 Nov 20;15(11):e0242187. doi: 10.1371/journal.pone.0242187 (PMC7679010; doi:10.1371/journal.pone.0242187)
Supplement: S3 Table — (DOCX) [file pone.0242187.s004.docx]

| **S3 Table: baseline characteristics, presented by cluster and by intervention allocation** | Cluster 1 | | | | | Cluster 2 | | | | Cluster 3 | | | | Cluster 4 | | | | Cluster 5 | | | |
| --- | --- | --- | --- | --- | --- | --- | --- | --- | --- | --- | --- | --- | --- | --- | --- | --- | --- | --- | --- | --- | --- |
|  | Intervention | | | Control | | Intervention | | Control | | Intervention | | Control | | Intervention | | Control | | Intervention | | Control | |
|  | N | | % | N | % | N | % | N | % | N | % | N | % | N | % | N | % | N | % | N | % |
| Maternal age | 31.2 | | 5.5 | 31.0 | 3.7 | 30.2 | 5.3 | 30.4 | 5.0 | 32.6 | 4.2 | 32.8 | 3.6 | 31.5 | 4.7 | 31.8 | 4.9 | 32.0 | 4.0 | 31.5 | 4.2 |
| Parity |  | |  |  |  |  |  |  |  |  |  |  |  |  |  |  |  |  |  |  |  |
| Primiparous | 36 | | 38.7% | 34 | 87.2% | 110 | 54.2% | 50 | 48.1% | 107 | 49.8% | 95 | 50.5% | 108 | 52.7% | 92 | 55.8% | 85 | 52.1% | 89 | 43.6% |
| Multiparous | 57 | | 61.3% | 5 | 12.8% | 93 | 45.8% | 54 | 51.9% | 108 | 50.2% | 93 | 49.5% | 97 | 47.3% | 73 | 44.2% | 78 | 47.9% | 115 | 56.4% |
| Cohabitation partners | | |  |  |  |  |  |  |  |  |  |  |  |  |  |  |  |  |  |  |  |
| Single | 4 | | 4.3% | 1 | 2.6% | 9 | 4.4% | 11 | 10.6% | 3 | 1.4% | 4 | 2.1% | 2 | 1.0% | 4 | 2.4% | 2 | 1.2% | 11 | 5.4% |
| Living together | 89 | | 95.7% | 38 | 97.4% | 194 | 95.6% | 93 | 89.4% | 212 | 98.6% | 184 | 97.9% | 203 | 99.0% | 161 | 97.6% | 161 | 98.8% | 193 | 94.6% |
| Immigrant status | | |  |  |  |  |  |  |  |  |  |  |  |  |  |  |  |  |  |  |  |
| Non-immigrant | 56 | | 60.2% | 30 | 78.9% | 103 | 51.2% | 29 | 28.2% | 142 | 67.9% | 132 | 70.6% | 162 | 79.0% | 107 | 64.8% | 132 | 83.5% | 170 | 83.3% |
| First generation | 17 | | 18.3% | 3 | 7.9% | 59 | 29.4% | 21 | 20.4% | 32 | 15.3% | 29 | 15.5% | 17 | 8.3% | 25 | 15.2% | 10 | 6.3% | 19 | 9.3% |
| Second generation | 20 | | 21.5% | 5 | 13.2% | 39 | 19.4% | 53 | 51.5% | 35 | 16.7% | 26 | 13.9% | 26 | 12.7% | 33 | 20.0% | 16 | 10.1% | 15 | 7.4% |
| Missing | 0 | | 0.0% | 1 | 2.6% | 2 | 1.0% | 1 | 1.0% | 6 | 2.8% | 1 | 0.5% | 0 | 0.0% | 0 | 0.0% | 5 | 3.1% | 0 | 0.0% |
| Health insurance | | |  |  |  |  |  |  |  |  |  |  |  |  |  |  |  |  |  |  |  |
| No | 0 | | 0.0% | 0 | 0.0% | 1 | 0.5% | 0 | 0.0% | 0 | 0.0% | 0 | 0.0% | 0 | 0.0% | 0 | 0.0% | 0 | 0.0% | 1 | 0.5% |
| Yes | 93 | | 100.0% | 39 | 100.0% | 202 | 99.5% | 104 | 100.0% | 215 | 100.0% | 188 | 100.0% | 205 | 100.0% | 165 | 100.0% | 163 | 100.0% | 203 | 99.5% |
| Education |  | |  |  |  |  |  |  |  |  |  |  |  |  |  |  |  |  |  |  |  |
| Lower | 8 | | 8.6% | 2 | 5.1% | 16 | 7.9% | 9 | 8.7% | 13 | 6.0% | 7 | 3.7% | 11 | 5.4% | 17 | 10.3% | 2 | 1.2% | 8 | 3.9% |
| Intermediate | 77 | | 82.8% | 31 | 79.5% | 138 | 68.0% | 81 | 77.9% | 95 | 44.2% | 75 | 39.9% | 145 | 70.7% | 115 | 69.7% | 83 | 50.9% | 126 | 61.8% |
| High | 8 | | 8.6% | 6 | 15.4% | 49 | 24.1% | 14 | 13.5% | 107 | 49.8% | 106 | 56.4% | 49 | 23.9% | 33 | 20.0% | 78 | 47.9% | 70 | 34.3% |
| Household income (euro/month) | | | |  |  |  |  |  |  |  |  |  |  |  |  |  |  |  |  |  |  |
| <1500 | 16 | | 17.2% | 1 | 2.6% | 27 | 13.3% | 34 | 32.7% | 17 | 7.9% | 9 | 4.8% | 17 | 8.3% | 22 | 13.3% | 18 | 11.0% | 29 | 14.2% |
| 1500-3000 | 43 | | 46.2% | 18 | 46.2% | 90 | 44.3% | 47 | 45.2% | 83 | 38.6% | 62 | 33.0% | 72 | 35.1% | 61 | 37.0% | 50 | 30.7% | 72 | 35.3% |
| >3000 | 34 | | 36.6% | 20 | 51.3% | 86 | 42.4% | 23 | 22.1% | 115 | 53.5% | 117 | 62.2% | 116 | 56.6% | 82 | 49.7% | 95 | 58.3% | 103 | 50.5% |
| Paid job (pregnancy) | | |  |  |  |  |  |  |  |  |  |  |  |  |  |  |  |  |  |  |  |
| No | 31 | | 33.3% | 6 | 15.4% | 64 | 31.5% | 37 | 35.6% | 38 | 17.7% | 26 | 13.8% | 40 | 19.5% | 42 | 25.5% | 28 | 17.2% | 34 | 16.7% |
| Yes | 62 | | 66.7% | 33 | 84.6% | 139 | 68.5% | 67 | 64.4% | 177 | 82.3% | 162 | 86.2% | 165 | 80.5% | 123 | 74.5% | 135 | 82.8% | 170 | 83.3% |
| Neighbourhood deprivation | | | |  |  |  |  |  |  |  |  |  |  |  |  |  |  |  |  |  |  |
| No | 88 | | 94.6% | 39 | 100.0% | 99 | 48.8% | 44 | 42.3% | 94 | 43.7% | 68 | 36.2% | 135 | 65.9% | 76 | 46.1% | 101 | 62.0% | 143 | 70.1% |
| Yes | 5 | | 5.4% | 0 | 0.0% | 104 | 51.2% | 60 | 57.7% | 121 | 56.3% | 120 | 63.8% | 70 | 34.1% | 89 | 53.9% | 62 | 38.0% | 61 | 29.9% |
| Smoking |  | |  |  |  |  |  |  |  |  |  |  |  |  |  |  |  |  |  |  |  |
| No | 76 | | 81.7% | 33 | 84.6% | 167 | 82.3% | 84 | 80.8% | 196 | 91.2% | 177 | 94.1% | 180 | 87.8% | 136 | 82.4% | 143 | 87.7% | 172 | 84.3% |
| Yes | 17 | | 18.3% | 6 | 15.4% | 36 | 17.7% | 20 | 19.2% | 19 | 8.8% | 11 | 5.9% | 25 | 12.2% | 29 | 17.6% | 20 | 12.3% | 32 | 15.7% |
| Alcohol |  | |  |  |  |  |  |  |  |  |  |  |  |  |  |  |  |  |  |  |  |
| No | 77 | | 82.8% | 33 | 84.6% | 170 | 83.7% | 91 | 87.5% | 163 | 75.8% | 147 | 78.2% | 170 | 82.9% | 140 | 84.8% | 113 | 69.3% | 154 | 75.5% |
| Yes | 16 | | 17.2% | 6 | 15.4% | 33 | 16.3% | 13 | 12.5% | 52 | 24.2% | 41 | 21.8% | 35 | 17.1% | 25 | 15.2% | 50 | 30.7% | 50 | 24.5% |
| Drugs |  | |  |  |  |  |  |  |  |  |  |  |  |  |  |  |  |  |  |  |  |
| No | 91 | | 97.8% | 39 | 100.0% | 201 | 99.0% | 98 | 94.2% | 214 | 99.5% | 187 | 99.5% | 204 | 99.5% | 162 | 98.2% | 157 | 96.3% | 203 | 99.5% |
| Yes | 2 | | 2.2% | 0 | 0.0% | 2 | 1.0% | 6 | 5.8% | 1 | 0.5% | 1 | 0.5% | 1 | 0.5% | 3 | 1.8% | 6 | 3.7% | 1 | 0.5% |
| Risk detected during pregnancy | | | |  |  |  |  |  |  |  |  |  |  |  |  |  |  |  |  |  |  |
| No | 45 | | 48.4% | 24 | 61.5% | 112 | 55.2% | 51 | 49.0% | 143 | 66.5% | 123 | 65.4% | 125 | 61.0% | 89 | 53.9% | 102 | 62.6% | 137 | 67.2% |
| Yes | 48 | | 51.6% | 15 | 38.5% | 91 | 44.8% | 53 | 51.0% | 72 | 33.5% | 65 | 34.6% | 80 | 39.0% | 76 | 46.1% | 61 | 37.4% | 67 | 32.8% |
| Indicated hours of maternity care | | | |  |  |  |  |  |  |  |  |  |  |  |  |  |  |  |  |  |  |
| 24-49 hours | | 57 | 61.3% | 30 | 85.7% | 156 | 80.3% | 53 | 51.0% | 180 | 87.4% | 163 | 87.6% | 168 | 87.0% | 144 | 90.0% | 131 | 81.9% | 181 | 94.3% |
| >49 hours | | 36 | 38.7% | 5 | 14.3% | 37 | 19.2% | 51 | 49.0% | 26 | 12.6% | 23 | 12.4% | 25 | 13.0% | 16 | 10.0% | 29 | 18.1% | 11 | 5.7% |
| Missing | | 0 | 0.0% | 4 | 10.3% | 10 | 4.9% | 0 | 0.0% | 9 | 4.2% | 2 | 1.1% | 12 | 5.9% | 5 | 3.0% | 3 | 1.8% | 12 | 5.9% |

Mean* with SD or number with % (presented as percentage of non-missing values). Missing value percentage of total.
