# Supplementary material for: Risk-guided maternity care to enhance maternal empowerment postpartum: A cluster randomized controlled trial
Source: PLoS One. 2020 Nov 20;15(11):e0242187. doi: 10.1371/journal.pone.0242187 (PMC7679010; doi:10.1371/journal.pone.0242187)
Supplement: S4 Table — (DOCX) [file pone.0242187.s005.docx]

| **S4 Table: Self-reported identified risk factors(s), by intervention allocation** | Intervention (n=879) | | Control (n=700) | |
| --- | --- | --- | --- | --- |
|  | N | % | N | % |
| Risk detected during pregnancy |  |  |  |  |
| No | 527 | 60.0% | 424 | 60.6% |
| Yes | 352 | 40.0% | 276 | 39.4% |
| Risk factors |  |  |  |  |
| Low empowerment sum-score | 52 | 15% | 64 | 23% |
| Alcohol during pregnancy | 186 | 53% | 135 | 49% |
| Smoking during pregnancy | 117 | 33% | 98 | 36% |
| Drugs during pregnancy | 12 | 3% | 11 | 4% |
| Use of medication | 3 | 1% | 2 | 1% |
| Combined alcohol, cigarette, and drugs use during the preconception period | 5 | 1% | 5 | 2% |
| Expected low empowerment regarding the baby | 96 | 27% | 75 | 27% |
| Anxiety disorders / depression | 96 | 27% | 68 | 25% |
| Fear for childbirth | 130 | 37% | 93 | 34% |
| Chronic disease unknown to obstetrician | 1 | 0% | 0 | 0% |
| Health literacy | 59 | 17% | 56 | 20% |
| Financial problems | 34 | 10% | 35 | 13% |
| Housing problems | 15 | 4% | 14 | 5% |
| No health insurance | 1 | 0% | 1 | 0% |
| Physical or emotional abuse | 1 | 0% | 1 | 0% |
| Problems family members | 4 | 1% | 4 | 1% |
