# Supplementary material for: Risk-guided maternity care to enhance maternal empowerment postpartum: A cluster randomized controlled trial
Source: PLoS One. 2020 Nov 20;15(11):e0242187. doi: 10.1371/journal.pone.0242187 (PMC7679010; doi:10.1371/journal.pone.0242187)
Supplement: S1 File — (PDF) [file pone.0242187.s006.pdf]

Lagendijk et al. BMC Pregnancy and Childbirth (2019) 19:4
https://doi.org/10.1186/s12884-018-2155-9

BMC Pregnancy and Childbirth

S1 File

STUDY PROTOCOL

Open Access

Client-tailored maternity care to increase maternal empowerment: cluster randomized controlled trial protocol; the healthy pregnancy 4 All-2 program

J. Lagendijk[1*], J. V. Been[1,2,3], H. E. Ernst-Smelt[1], G. J. Bonsel[4], L. C. M. Bertens[1] and E. A. P. Steegers[1]

Abstract

Background: The postpartum period is an important period for preventive strategies as common maternal and child health risks may become manifest. Women with a lower socioeconomic status tend to have lower maternal empowerment. Increasing their risks of adverse maternal and child health outcomes. This study aims to assess the effectiveness of a primary care level intervention. Delivered to maternity care assistants, aiming to increase maternal empowerment postpartum.

Methods: This study is part of the Dutch nationwide "Healthy Pregnancy 4 All-2" (HP4All-2) program, which aims to identify vulnerable mothers and young children at risk of adverse health outcomes, and subsequently improve their care. This program targets women from deprived neighborhoods.
A pragmatic cluster randomized controlled trial will be undertaken in 12 maternity care organizations. Maternity care organizations in urban municipalities (i.e. the clusters) will be randomized to either a systematic risk assessment during pregnancy with emphasis on identification of non-medical risk factors for adverse maternal and neonatal health outcomes, and subsequent adaptation of care towards a client-tailored approach during pregnancy and the postpartum period, or solely the systematic risk assessment. The primary outcome is the prevalence of a low maternal empowerment score postpartum. Secondary maternal outcomes cover health-related quality of life, postnatal depression, smoking, alcohol consumption, illicit drug use. Finally, maternal and neonatal health care utilization postpartum are recorded. All outcomes will be analyzed according to the intention-to-treat principle, using multi-level mixed effects models.

Discussion: The study will contribute to evidence regarding the effectiveness of client-tailored, risk-based maternity care to increase maternal empowerment postpartum.

Trial registration: Netherlands Trial Registry (NTR) 6311, registered 03-27-2017.

Keywords: Maternity care, Health inequalities, Risk factors, Empowerment

* Correspondence: j.lagendijk.2@erasmusmc.nl
[1]Department of Obstetrics and Gynecology, Erasmus MC, University Medical Centre Rotterdam, PO Box 2040, 3000, CA, Rotterdam, The Netherlands
Full list of author information is available at the end of the article

© The Author(s). 2019 **Open Access** This article is distributed under the terms of the Creative Commons Attribution 4.0 International License (http://creativecommons.org/licenses/by/4.0/), which permits unrestricted use, distribution, and reproduction in any medium, provided you give appropriate credit to the original author(s) and the source, provide a link to the Creative Commons license, and indicate if changes were made. The Creative Commons Public Domain Dedication waiver (http://creativecommons.org/publicdomain/zero/1.0/) applies to the data made available in this article, unless otherwise stated.

Introduction

The postpartum period, defined by the World Health Organization (WHO) as the period from childbirth to the 42nd day following delivery, is an important period for preventive strategies as common maternal and child health problems become apparent. Health problems include maternal and neonatal infections, postpartum hemorrhage, neonatal respiratory and feeding problems, and psychological problems of varying severity [1]. The purpose of maternity care provided during this period is manifold; 1) to promote physical health of the mother and her baby, 2) to promote coping of parents with the new situation through providing support, and 3) to promote parental empowerment in handling their baby [1, 2]. To achieve this, maternity care consists not only of medical care, but also of psychosocial care, support, and education. Maternity care assistants (MCAs) provide care following delivery at home on a daily basis for over a week (Table 1).

Women with a lower socioeconomic status (SES) are more at risk of developing adverse maternal and child health outcomes within both western and non-western countries. The resulting inequity in maternal and child health outcomes already starts before birth and extends into early childhood [3–7]. In addition, women with a lower SES tend to have a lower empowerment as compared to women with a higher SES [8]. The WHO defined empowerment as a process through which people gain greater control over decisions and actions affecting their health [9]. Maternal empowerment is a valuable outcome per se, but also an important predictor of maternal and child health and increased health care utilization [10–13]. Thus, the already increased risk of adverse health outcomes among women with a low SES may be further augmented by insufficient maternal empowerment.

In the Netherlands, a national protocol is used during pregnancy to assess the need for maternity care postpartum, expressed in hours of care [14]. This Dutch national maternity care indication protocol (abbreviated by 'LIP' in Dutch) is in line with the assessment by midwives and obstetricians, and mostly focuses on medical risk factors (Table 1). This protocol, however, insufficiently acknowledges the relevance of non-medical risk factors, including those associated with low SES, for health outcomes around birth and the associated additional need for maternity care. Our study amended the existing risk assessment with non-medical risk factors, and introduced tailored care adaptations according to the risk profile. The intensive and preventive structure of maternity care along with the opportunities for maternity care assistants (MCAs) to tailor care from pregnancy onwards creates a window of opportunity for patient-tailored care (Table 2). We hypothesize that the delivery of client-tailored, risk-guided maternity care could improve maternal empowerment and thereby reduce the probability of risk factors developing into manifest problems [15, 16].

This study is part of the Dutch nationwide "Healthy Pregnancy 4 All-2" (HP4All-2) program, which aims to improve the identification of and care for mothers and young children at risk of adverse health outcomes [17]. HP4All-2 particularly targets women from deprived neighborhoods. In the current cluster randomized controlled trial (C-RCT) we aim to specifically address health inequalities during the postpartum period by 1)

Table 1 The Dutch perinatal care system

Dutch perinatal care system
Antenatal care in The Netherlands is based on the concept that pregnancy, childbirth, and the postpartum period are fundamentally physiologic processes. Obstetric risk selection is performed by community midwives or obstetricians and is based on the 'List of Obstetric Indications' (LOI), which specifies manifest conditions that define a low, medium, or high-risk pregnancy. An obstetrician will care for women with a high-risk pregnancy whereas a community midwife may provide care to women with a low or a medium risk.<br>Based on the LOI approximately 30% of all pregnant women are considered to have a low risk throughout their pregnancy and delivery. In 2015, 13.1% of all births in the Netherlands were home births. In case of an uncomplicated institutional delivery the mother will usually be discharged home within a few hours. Regardless of the risk indication based on the LOI, the community midwife will be responsible for care of the mother when discharged home during the postpartum period.<br>Maternity care is provided by maternity care assistants (MCAs) and will start at home, or – less frequently – in a primary care birth center, under supervision of the community midwife. Following delivery, a MCA visits and supports the family at home on a daily basis for the first eight to ten consecutive days. Initially maternity care covers six to eight hours a day but this is tapered off towards the end of the care period. If a mother is hospitalized after delivery for a longer duration, the provided care by MCAs is reduced. However, based on specific indications (see Table 2) the care provision by MCAs may be expanded.

Table 2 Maternity care organizations

Maternity care organizations
There are around 120 maternity care organizations in the Netherlands that function as independent enterprises. Women can sign up during pregnancy with any maternity care organization that provides care in their neighborhood. On average, 95% of all women make use of some amount of maternity care.<br>During pregnancy a MCA will assess a woman's expected care requirements during a scheduled home visit around 25–37 weeks of gestation. For primiparous women this intake is scheduled as a home visit, whereas for multiparous women this intake is conducted per telephone. Compensation from health insurers to maternity care organizations differs according to this policy. The intensity of care provision during the postpartum period is based on the indications denoted in the Dutch national indication protocol (abbreviated by LIP in Dutch). Examples of indications that add to the intensity of care are: not being physically self-sufficient, having a psychological illness, and having other children under the age of four years. An example of an indication that will downscale the intensity of care is planning to bottle feed rather than breastfeed the newborn.<br>The minimum volume of care at home is set at 24 h over eight days, the recommended volume is set at 49 h, and its maximum amount is set at 80 h, depending on specific indications, spread out over eight to ten days. Maternity care is covered by the general health care insurance (which is mandatory for every Dutch inhabitant) with exception of an out-of-pocket payment of €4.30 per hour (2017).

systematically improving identification of non-medical risk factors during pregnancy, and 2) using this risk profile to provide client-tailored care during pregnancy and the postpartum period.

Methods

We designed a pragmatic C-RCT in six municipalities in the Netherlands to assess the effectiveness of a complex intervention to promote maternal empowerment in the postpartum period. The intervention under study consists of a systematic risk assessment during pregnancy, with emphasis on identifying non-medical risk factors for adverse maternal and neonatal health outcomes, in conjunction with client-tailored care during pregnancy and the postpartum period based on the obtained risk profile. We used the SPIRIT statement for clinical trial protocols to guide reporting of our protocol [18].

Study setting

This study is embedded in the HP4All-2 program that aims to reduce perinatal health inequalities and to improve the care for young children and their mothers [17]. Six out of ten participating municipalities within the overall HP4All-2 program participate in this trial. A detailed description of the selection process of municipalities has been published before [17]. We designed a two-arm C-RCT in six municipalities, including 12 maternity care organizations (Fig. 1). Two adjacent municipalities were merged to ensure that enough eligible women could be expected to be included during the study period. The resulting five participating municipalities were each subdivided into an intervention cluster and a control cluster, resulting in a total number of ten clusters. Each cluster consisted of one or more distinct maternity care organizations, depending on the expected number of eligible women within each organization (Fig. 1). Clustering at the level of the maternity care organization was chosen to avoid contamination of the intervention, which includes education of MCAs, between MCAs within maternity care organizations and between mother-baby dyads cared for by the same MCA. Outcomes will be assessed at the individual level.

Eligibility criteria

Criteria for maternity care organizations

Participation of maternity care organizations is based on several factors: their willingness to participate, their capacity (defined as the expected number of eligible women to be cared for during the study period), and their ability to allow a sufficient number of MCAs to participate throughout the study period (sufficiency is based on the expected number of participants included). To increase cooperation of the participating organizations in the study, we aligned our protocol with their standard working schedules. Such an alignment for example would allow that the timing of our intervention, which starts during a scheduled home visit, differs regarding weeks of gestation.

Criteria for health care professionals

MCAs from the participating organizations will act as local researchers after completing the training sessions.

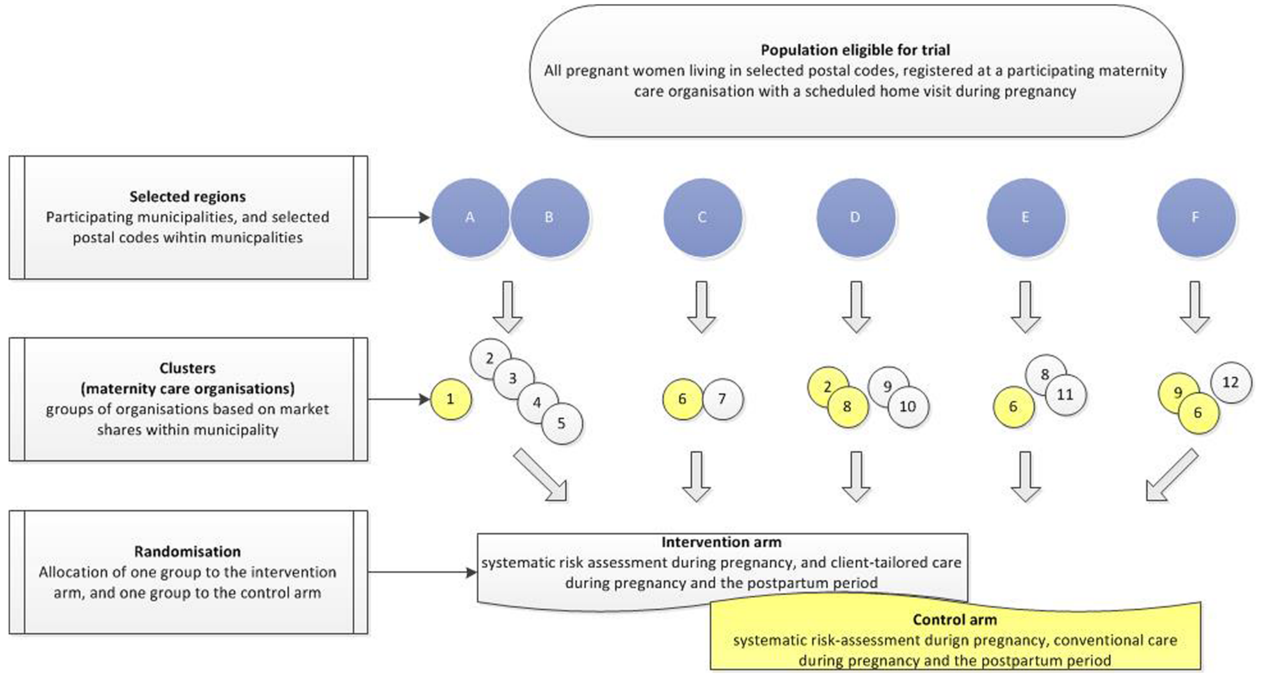

Fig. 1 Trial profile

They will inform potential participants about the study and obtain written informed consent. They also serve as the main point of contact between the research team and the participants, and have a key role in sustaining participant recruitment and participant participation throughout the study period.

Exclusion criteria for maternity care organizations

Inability to conduct a scheduled home visit for all participants.

Inclusion criteria participants

All pregnant women to be cared for by participating maternity care organizations, with a scheduled home visit during pregnancy, are eligible and invited to take part in the study.

Exclusion criteria participants

Unwillingness to sign a written informed consent form.

Intervention

Intervention at the level of the participant

The intervention consists of two dimensions: 1) a systematic risk assessment during pregnancy with emphasis on identification of non-medical risk factors for adverse maternal and neonatal health outcomes, and 2) subsequent adaptation of maternity care towards a client-tailored approach during pregnancy and the postpartum period.

The first part is based on an electronic risk assessment questionnaire and covers six different domains related to individual level SES and psychological factors. All assessed factors are known to be associated with adverse health outcomes during pregnancy, childbirth or early childhood. The assessment consists of three main components: 1) the validated Mind2Care (M2C) tool [19], 2) the Maternal Empowerment Questionnaire (MEQ) [20] with slight modifications, and (3) eight additional questions regarding a person's financial situation, whether they have health care insurance, and their use of health care in the year prior to pregnancy. Two out of five domains of the MEQ were omitted for this assessment after consulting the authors (i.e. domains "the future" and "maternity care") as being not applicable. All other questions were grammatically adjusted for application during pregnancy, rather than after childbirth.

The MCA will review and discuss the identified risk factor(s) upon completion of the electronic assessment by the participant. Specific risk factors are linked to specific care pathways, which guide the MCA into taking appropriate action, and organize client-tailored care during pregnancy and the postpartum period. Care pathways consist of different steps a MCA can take to initiate a risk-guided provision of care in collaboration with professionals from different echelons. These steps can include communication with an involved community midwife or obstetrician but also with professionals from the public health sector. Care pathways are tailored in order to accommodate practice variations within maternity care organizations to fit local protocols and habits.

Aspects relevant to both intervention and control arms

Three large health insurers in the Netherlands agreed to additional fund a home risk intake (instead of per telephone call) for all study participants rather than for primiparous women alone. Prior to inclusion of participants, MCAs from both the intervention and the control arm will be trained extensively to use the renewed risk assessment. Each pair of clusters within a municipality will be trained together. The training consists of two sessions of three hours and will be led by a well-established Dutch educational agency together with the executing investigator (JL). For each cluster, the first training will take place at the start of the study and the second refresher training will be scheduled three months after the start.

The training program is focused on providing various communication skills for health care professionals that will build their confidence to address risks within a participant's social situation and lifestyle (the non-medical risk component). The following key topics were covered during the training program: creating opportunities for constructive conversations, empowering women to take control and manage their personal situation, and asking additional questions to identify the need of a participant regarding her health care provision. In addition, this training program covers group exercises to enhance effective and respectful engagement, in order to correctly include participants in a research trial, and in order to answer participants' questions regarding the study protocol effectively.

The second training was set up to refresh the knowledge and skills obtained in the first training and to evaluate the feasibility of the study protocol. If deemed necessary by the investigator team, minor adjustments in study protocol may be made to address any issues raised. Prior to the start of the study, all staff working in the administrative or the management sector of every participating organization receives training by the executing investigator of the research team (JL). This training focuses on the study protocol and all administrative tasks related to participant engagement with the study (i.e. sending the study flyer prior to the intake, scheduling the intake and informing eligible women on the study; see "participant timeline").

Intervention on the level of the MCA and the level of maternity organizations

Prior to the start of the intervention during the postpartum period, all MCAs working for maternity care organizations

allocated to the intervention arm receive an additional three-hour training session directed towards the subsequent step of a tailored care approach, once the risk profile is known. This training session is led by another well-established Dutch educational agency together with the executing investigator of the research team (JL).

The content of this training includes the rationale for the study; background information highlighting the importance of the social gradient in perinatal and postpartum health, a group conversation of the contribution of the risk factors evaluated to this gradient, and skills training. Skills training is focused on different topics: shaping knowledge of the social gradient in health to create and encourage a preventive approach in care, creating confidence to address a participant's social situation and lifestyle, and skills to increase practical and emotional social support. In addition, the timing of the obtained skills during the early postpartum period and the subsequent preventive approaches will be discussed in-group exercises. The obtained skills should strengthen a participant's own capabilities in taking care of herself, her baby, and her family, thereby increasing maternal empowerment.

Control situation

To adequately compare the intervention and the control group with regard to their medical and non-medical risk factors during pregnancy, the risk assessment during pregnancy is performed in both study arms. The MCA in the control arm is however instructed not to discuss the detected risks with the participant upon completion of the risk assessment or to discuss the findings with other health care professionals. One exception to this blinding of participants is made for the risk factor "suicidal thoughts", given the severity of this risk identification. In the control group the risk assessment will be followed by conventional care during pregnancy and the postpartum period.

Outcomes

Primary outcome

The primary outcome is the prevalence of a low maternal empowerment score postpartum, defined as a score beneath the 20th centile of all empowerment scores within the control arm (Table 3). Maternal empowerment will be assessed via the MEQ at the last day of the provided maternity care, usually at day eight after childbirth, and will be defined as the median score across four out of five domains within the MEQ (i.e. scores from the domain 'Maternity care' will not be considered for the primary outcome (Table 3)) [20].

Secondary outcomes

Secondary outcome measures address maternal health-related quality of life, maternal depression, maternal smoking, alcohol consumption, drug use, and maternal and neonatal health care utilization postpartum. All individual outcome measures are summarized in Table 3 with used definitions and timing of assessment.

The adherence to the study protocol within the intervention arm will be assessed quantitatively using questionnaires filled out by the MCAs who provided the care in the early postpartum period. This questionnaire will be filled out for each participant separately and assesses the MCAs' knowledge of the maternal risk factors obtained during pregnancy and their knowledge of the applied client-tailored care during pregnancy and the postpartum period.

In addition we will assess the effectiveness of the implementation process and the adherence to the study protocol in both arms. The effectiveness of the implementation process within maternity care organizations will be assessed quantitatively with a questionnaire addressed to professionals working in the administrative or management sector of each maternity care organization.

Participant timeline

Potentially eligible women receive a study flyer when registering with one of the participating maternity care organizations. During a scheduled home visit (i.e. the intake), the participant will fill out the structured risk assessment electronically. At the end of the standard maternity care provision period, a second questionnaire will be filled out assessing the primary outcome and a number of secondary outcomes (Table 3). A third electronic questionnaire will be sent to participants during the late postpartum period (i.e. six weeks after they gave birth). This questionnaire will assess multiple secondary outcomes (Table 3).

Sample size

Calculation of sample size is based on the presumed effect of the intervention on the primary outcome at the individual level. The intervention is considered to be effective when the prevalence of a low empowerment score, is reduced by 50%; in other words, our sample size is calculated to determine a proportion difference from a low empowerment score at the 20th centile towards the 10th centile.

A previous study using the MEQ, in an unselected population of 2675 women in the postpartum period, showed a mean score over four domains of 3.70, with a left-skewed distribution (25th centile 3.46 and 75th centile 3.88) [20]. We hypothesize that the intervention will lead to a decline of an empowerment score of < 3.38 (i.e. the 20th centile) by 50%. At an alpha of 0.05 and 80% power, we will require 196 participants per arm and as such 392 participants in total.

Table 3 Primary and all secondary outcomes at the individual level with definitions and timing of assessment

Outcomes	Postpartum period	
	Early (1–2 weeks after childbirth)	Late (6–12 weeks after childbirth)
Primary	*Maternal empowerment*	
Definition	Low empowerment score (no/yes) defined as a score beneath the 20th centile within the control group	
Assessment	Maternal Empowerment Questionnaire (MEQ). Overall scoring based on the median score across the following four domains; "Looking after yourself", "My baby", "My family", "The future". Values per question: 1 "Never" 2 "Sometimes", 3 "Usually", and 4 "Always".	
Secondary	*Maternal health related quality of life*	*Maternal depression (postnatal depression)*
Definition	Continuous outcome ranging from zero (dead) to one (full health).	Dichotomous outcome based on the sum score: "No" sum score < 13 and "Yes" sum score > 12.
Assessment	5-level EQ-5D (EQ-5D-5 L). Each dimension within the questionnaire has 5 levels: 1 "no problems", 2 "slight problems", 3 "moderate problems", 4 "severe problems", and 5 "extreme problems". Resulting in health profiles ranging from "11,111" through "55,555". Continuous score calculated with the obtained profiles of the questionnaire with the validated EQ-5D-5 L calculator.	Edinburgh Postnatal Depression Scale (EPDS). Ten item scale. Responses are scored 0, 1, 2 and 3 based on the seriousness of the symptom. Range 0–30.
	Maternal perceived health	*Maternal health care utilization*
Definition	Continuous outcome ranging from zero (the worst health possible) to 100 (the best health possible).	Categorical outcome: "No additional care", "One visit to the A and E department, GP, or GP out-of-hours service", "Multiple visits", and "Admission in a hospital"
Assessment	EuroQol-visual analogue scales (EQ-VAS) represented by a 20 cm vertical scale.	Q1: Since your baby was born, have you had any symptoms for which you have been to the accident and emergency (A and E) department, GP or out-of-hours GP service?
		Q2: Have you been admitted to hospital since your baby was born?
		Neonatal health care utilization
Definition		Categorical outcome ranging from: "No additional care", "One visit to the A and E department, GP, or GP out-of-hours service", "Multiple visits", and "Admission in a hospital"
Assessment		Q1: Have you been to the accident and emergency (A and E) department, GP or out-of-hours GP service for your baby since he or she was born?
		Q2: Has your baby been admitted to hospital since he or she was born?
	Maternal cigarette use	*Maternal cigarette use*
Definition	Dichotomous outcome (no/yes): "Yes" defined as any usage	Dichotomous outcome (no/yes): "Yes" defined as any usage
Assessment	Q: Do you smoke?	Q: Do you smoke?
	Maternal alcohol use	*Maternal alcohol use*
Definition	Dichotomous outcome (no/yes): "Yes" defined as any usage	Dichotomous outcome (no/yes): "Yes" defined as any usage
Assessment	Q: Do you drink alcohol?	Q: Do you drink alcohol?
	Maternal drugs use	*Maternal drugs use*
Definition	Dichotomous outcome (no/yes): "Yes" defined as any usage	Dichotomous outcome (no/yes): "Yes" defined as any usage
Assessment	Q: Do you use drugs? Marijuana, hash and weed are drugs too.	Q: Do you use drugs? Marijuana, hash and weed are drugs too.

The intervention will be implemented at the cluster level (i.e. (groups of) maternity care organizations), while the intervention effect will be assessed at the participant level. We expect a small variance in provided care between clusters as is common in primary care practices [21]. The Intraclass Correlation Coefficient (ICC) is therefore set at 0.05. The Variance Inflation Factor (VIF), calculated with the formula of Donner et al. [22], is calculated to be 2.91.

We account for a lost to follow-up of 33% (i.e. from inclusion to assessment of primary outcome). The target sample size is therefore set at 856 (i.e. 1.5*(2.91*196)) participants per arm.

Recruitment
To achieve adequate participant enrolment, an inclusion target per month will be set for each organization. Monitoring of this target will be communicated on monthly bases with all local researchers. Not achieving this target is seen as the impediment of effective recruitment and action will be taken to improve the effectiveness in recruiting eligible participants. An example of such action would be evaluating the barriers for participant inclusion, evaluating engagement barriers, and providing additional support for MCAs.

Randomization and allocation
We will perform allocation by clusters, with the maternity care organization(s) as the randomization unit, to minimize possible contamination effects among professionals. Each cluster was randomized to the intervention arm or the control arm by a statistician not involved in the implementation of the trial and blind to the identity of the clusters and the incorporated maternity care organizations.

Blinding
Maternity care organizations agreed to participate, and to accept any allocation, prior to the randomization procedure.

Study information to participants strictly contains information related to the allocated arm, without mentioning the randomized design of the trial or the changes in the provided care during pregnancy and the postpartum period.

Data collection
No interim analyses are planned, and all outcomes will be analyzed following data collection.

Statistical methods
All analyses will be performed according to the intention-to-treat principle.

Descriptive statistics will be presented, and formal inference will be based on hypothesis testing with two-sided statistical significance assessed at the 5% level.

Multiple imputation using chained equations will be used to account for missing data in baseline characteristics. We will analyze the effect of the intervention on our primary and secondary outcome measurements using multilevel mixed-effects linear or logistic regression analyses with an assumed random effect for each cluster.

Data monitoring
A data monitoring committee (DMC) is not installed, as the ethics committee deemed it as unnecessary, because the risks for participants are negligible. All aspects of the intervention fall within the scope of conventional maternity care.

The funding source has no role in the design of the study, and neither in the collection and management of the data.

Discussion
With this C-RCT we aim to systematically increase the identification of non-medical risk factors during pregnancy, and use the obtained risk profile for improved client-tailored care during the postpartum period. We envisage that this intervention creates an additional opportunity for MCAs to empower women during the postpartum period. Increased maternal empowerment has the potential to enhance their self-efficacy, their quality of life, and their child's well-being during the postpartum period [10, 13].

To our knowledge this is the first multicenter trial of client-tailored, risk-guided maternity care, creating a unique opportunity to investigate its potential to improve maternal empowerment. In concordance with the overall aim of the HP4All-2 program, this study targets women from deprived neighborhoods, aiming to reduce adverse maternal and neonatal outcomes of those who are at greatest risk.

There are several challenges in the execution of this complex C-RCT that merit discussion. This C-RCT is embedded in a complex existing collaborative setting involving multiple municipalities and maternity care organizations and their professionals. Particular challenges include maintaining engagement in 17 sites spread across the country, engaging staff groups employed by different organizations, and motivating MCAs from different organizations to change their routine working practices and adapt to a more risk-guided provision of care. In addition, there is an unequivocal registration of the personalized care undertaken by maternity care professionals during the postpartum.

To conclude, this C-RCT is the first trial in the Netherlands aiming to empower women during the postpartum period. Providing a risk assessment, with emphasis on non-medical risks related to SES and psychopathology, facilitates client-tailored care and may contribute to prevention of adverse health outcomes for mother and child.

Abbreviations
C-RCT: Cluster Randomized Controlled Trial; HP4All-2: Healthy Pregnancy 4 All-2; ICC : Intraclass Correlation Coefficient; MCA: Maternity Care Assistants; MEQ: Maternal Empowerment Questionnaire; SES: Socioeconomic status; VIF: Variance Inflation Factor

Acknowledgements
Not applicable.

Funding
This study is funded by the Ministry of Health, Welfare and Sports (grant number: 323911). JVB is funded by personal fellowships from the Erasmus MC and the Netherlands Lung Foundation (4.2.14.063JO).
The study protocol has undergone peer-review by the funding body. The funding sources had no role in the design of this study and will not have any role during its execution, analyses, interpretation of the data, or decision to submit results.

Availability of data and materials
This manuscript consists of a study protocol. After data collection and management, all data will be made available on request.

Authors' contributions
Substantial contributions to the conception or design of the work were made by JL, JVB, HEE, GJB, and EAS. EAS and GJB contributed to the acquisition. Analysis were performed by JL and JVB, who drafted the work too. The interpretation of data for the work was performed by JL, JVB, LCB, EAS. All authors revised the work critically for important intellectual content, and gave final approval of the version to be published.

Ethics approval and consent to participate
We have obtained multi-site approval from the Daily Board of the Medical Ethics Committee Erasmus MC (METC 2015–156). Ethical approval to collaborate with the board of each municipality and with the board of maternity care organizations was provided. In addition we obtained written informed consent from all three levels of participants. Written informed consent was obtained from every participant prior to inclusion. In addition, we obtained written informed consent from the board of every participating municipality (Rotterdam, Schiedam, Groningen, Utrecht, Almere, and Arnhem), and from the board of every participating maternity care organization (Kraamzorg Rotterdam, Careyn Kraamzorg, Kraamzorg Sara, Kraamzorg de Eilanden, Kraamzorg de Bakermand, Kraamzorg het Groene Kruis, Kraamvogel, Zin kraamzorg, Yunio, KraamInzicht, de Waarden, and Liemerscare).

Consent for publication
Not applicable.

Competing interests
The authors declare that they have no competing interests.

Publisher's Note

Springer Nature remains neutral with regard to jurisdictional claims in published maps and institutional affiliations.

Author details
[1]Department of Obstetrics and Gynecology, Erasmus MC, University Medical Centre Rotterdam, PO Box 2040, 3000, CA, Rotterdam, The Netherlands. [2]Division of Neonatology, Department of Pediatrics, Erasmus MC, University Medical Centre Rotterdam, PO Box 2040, 3000, CA, Rotterdam, the Netherlands. [3]Department of Public Health, Erasmus MC, University Medical Centre Rotterdam, PO Box 2040, 3000, CA, Rotterdam, the Netherlands. [4]Department of Obstetrics and Gynecology, University Medical Centre Utrecht, PO Box 85090, 3508, AB, Utrecht, The Netherlands.

Received: 27 December 2017 Accepted: 17 December 2018
Published online: 03 January 2019

References

1. Data WLC-i-P: WHO recommendations on postnatal care of the mother and newborn. 2013.
2. T.A. W. Adjusting to motherhood maternity care assistance during the postpartum period: how to help new mothers cope. J Neonatal Nurs. 2006;12(5):163–71.
3. Steegers EA, Been JV. Health inequalities: congenital or preventable? Ned Tijdschr Geneeskd. 2015;160(0):D83.
4. Timmermans S, Bonsel GJ, Steegers-Theunissen RP, Mackenbach JP, Steyerberg EW, Raat H, Verbrugh HA, Tiemeier HW, Hofman A, Birnie E, et al. Individual accumulation of heterogeneous risks explains perinatal inequalities within deprived neighbourhoods. Eur J Epidemiol. 2011;26(2):165–80.
5. Mackenbach JP. The persistence of health inequalities in modern welfare states: the explanation of a paradox. Soc Sci Med. 2012;75(4):761–9.
6. Vos AA, Posthumus AG, Bonsel GJ, Steegers EA, Denktas S. Deprived neighborhoods and adverse perinatal outcome: a systematic review and meta-analysis. Acta Obstet Gynecol Scand. 2014;93(8):727–40.
7. Steegers EA, Barker ME, Steegers-Theunissen RP, Williams MA. Societal valorisation of new knowledge to improve perinatal health: time to act. Paediatr Perinat Epidemiol. 2016;30(2):201–4.
8. Ahmed S, Creanga AA, Gillespie DG, Tsui AO. Economic status, education and empowerment: implications for maternal health service utilization in developing countries. PLoS One. 2010;5(6):e11190.
9. (WHO) WHO: Health Promotion Glossary. *WHO/HPR/HEP/981* 1998.
10. Carlson GJ, Kordas K, Murray-Kolb LE. Associations between women's autonomy and child nutritional status: a review of the literature. Matern Child Nutr. 2015;11(4):452–82.
11. Hou X, Ma N. The effect of women's decision-making power on maternal health services uptake: evidence from Pakistan. Health Policy Plan. 2013; 28(2):176–84.
12. Bloom SS, Wypij D, Das Gupta M. Dimensions of women's autonomy and the influence on maternal health care utilization in a north Indian city. Demography. 2001;38(1):67–78.
13. Pratley P. Associations between quantitative measures of women's empowerment and access to care and health status for mothers and their children: a systematic review of evidence from the developing world. Soc Sci Med. 2016;169:119–31.
14. (KNOV) KNOvV: Instrument voor toekenning van kraamzorg: Partusassistentie en kraamzorg gedurende de kraamperiode.
15. N W: What is the evidence on effectiveness of empowerment to improve health? WHO Regional Office for Europe Health Evidence Network report. 2006. http://www.euro.who.int/__data/assets/pdf_file/0010/74656/E88086.pdf. Accessed 1 Feb 2006.
16. Sado L, Spaho A, Hotchkiss DR. The influence of women's empowerment on maternal health care utilization: evidence from Albania. Soc Sci Med. 2014;114:169–77.
17. Waelput AJM, Sijpkens MK, Lagendijk J, van Minde MRC, Raat H, Ernst-Smelt HE, de Kroon MLA, Rosman AN, Been JV, LCM B, et al. Geographical differences in perinatal health and child welfare in the Netherlands: rationale for the healthy pregnancy 4 all-2 program. BMC Pregnancy Childbirth. 2017;17(1):254.
18. Chan AW, Tetzlaff JM, Altman DG, Laupacis A, Gotzsche PC, Krle AJK, Hrobjartsson A, Mann H, Dickersin K, Berlin JA, et al. SPIRIT 2013 statement: defining standard protocol items for clinical trials. Rev Panam Salud Publica. 2015;38(6):506–14.
19. Quispel C, Schneider TA, Bonsel GJ, Lambregtse-van den Berg MP. An innovative screen-and-advice model for psychopathology and psychosocial problems among urban pregnant women: an exploratory study. J Psychosom Obstet Gynaecol. 2012;33(1):7–14.
20. Scheerhagen M, van Stel HF, Tholhuijsen DJ, Birnie E, Franx A, Bonsel GJ. Applicability of the ReproQ client experiences questionnaire for quality improvement in maternity care. PeerJ. 2016;4:e2092.
21. Adams G, Gulliford MC, Ukoumunne OC, Eldridge S, Chinn S, Campbell MJ. Patterns of intra-cluster correlation from primary care research to inform study design and analysis. J Clin Epidemiol. 2004;57(8):785–94.
22. Donner A, Birkett N, Buck C. Randomization by cluster. Sample size requirements and analysis. Am J Epidemiol. 1981;114(6):906–14.

Ready to submit your research? Choose BMC and benefit from:

- fast, convenient online submission
- thorough peer review by experienced researchers in your field
- rapid publication on acceptance
- support for research data, including large and complex data types
- gold Open Access which fosters wider collaboration and increased citations
- maximum visibility for your research: over 100M website views per year

At BMC, research is always in progress.

Learn more biomedcentral.com/submissions

BMC
